# The prevalence of mental health problems in sub-Saharan adolescents: A systematic review

**DOI:** 10.1371/journal.pone.0251689

**Published:** 2021-05-14

**Authors:** Astrid Jörns-Presentati, Ann-Kathrin Napp, Anja S. Dessauvagie, Dan J. Stein, Deborah Jonker, Elsie Breet, Weslin Charles, Renier L. Swart, Mari Lahti, Sharain Suliman, Ronelle Jansen, Leigh L. van den Heuvel, Soraya Seedat, Gunter Groen

**Affiliations:** 1 Department of Social Work, University of Applied Sciences, Hamburg, Germany; 2 Department of Psychiatry and Neuroscience Institute, SAMRC Unit on Risk & Resilience in Mental Disorders, University of Cape Town, Cape Town, Western Cape, South Africa; 3 Department of Psychiatry and Mental Health, University of Cape Town, Cape Town, Western Cape, South Africa; 4 Department of Health and Well-being, Turku University of Applied Sciences, Turku, Province of Western Finland, Finland; 5 Department of Psychiatry, SAMRC Unit on the Genomics of Brain Disorders and SARChI Chair in Posttraumatic Stress Disorder, Stellenbosch University, Cape Town, Western Cape, South Africa; 6 School of Nursing, University of the Free State, Bloemfontein, Free State Province, South Africa; Koc University School of Medicine, TURKEY

## Abstract

**Background and purpose:**

Most research regarding child and adolescent mental health prevention and promotion in low-and middle-income countries is undertaken in high-income countries. This systematic review set out to synthesise findings from epidemiological studies, published between 2008 and 2020, documenting the prevalence of mental health problems in adolescents from across sub-Saharan Africa.

**Methods:**

A systematic search of multiple databases (MEDLINE, PsycINFO, Scopus) and Google Scholar was conducted guided by the Joanna Briggs Institute (JBI) Reviewer’s manual for systematic reviews of observational epidemiological studies. Studies included reported prevalence outcomes for adolescents aged 10–19 using either clinical interviews or standardized questionnaires to assess psychopathology. Clinical samples were excluded.

**Results:**

The search yielded 1 549 records of which 316 studies were assessed for eligibility and 51 met the inclusion criteria. We present a qualitative synthesis of 37 of these 51 included articles. The other 14 studies reporting prevalence rates for adolescents living with HIV are published elsewhere. The prevalence of depression, anxiety disorders, emotional and behavioural difficulties, posttraumatic stress and suicidal behaviour in the general adolescent population and selected at-risk groups in 16 sub-Saharan countries (with a total population of 97 616 adolescents) are reported.

## Introduction

Worldwide, mental disorders have a high prevalence, chronicity and morbidity [1]. Adolescent populations are especially vulnerable to developing mental disorders [2] and contribute to the high burden of mental and substance use disorders globally [3]. In sub-Saharan Africa, 23% of the population comprise of adolescents between 10–19 years of age, making up the greatest proportion of the population [4].

Despite the fact that adolescents living in low-and middle-income countries (LIMC) are disproportionately affected, there are limited data on the prevalence of adolescent mental disorders in sub-Saharan Africa [5–10] with most research informing child and adolescent mental health promotion in LMIC being undertaken in high-income countries (HIC) [6]. In a recent systematic review and meta-analysis of common mental disorders measured with the General Health Questionnaire only one study from Nigeria was included [11]. There is a pressing need to intensify local research capacity in LMIC [7, 9].

It is estimated that 10–20% of children and adolescents experience mental health problems worldwide [5, 12]. The most recent systematic review focusing solely on the prevalence of child mental health problems in sub-Saharan Africa reported that one in seven children and adolescents (14.3%) experiences significant psychological challenges, and one in ten (9.5%) qualifies for a psychiatric diagnosis [13]. Included studies showed that adolescent mental health morbidity is contributed to by a combination of impoverished living conditions, a high prevalence of HIV and AIDS, in addition to risk factors for mental disorders that are common in high-income countries (e.g. paternal psychopathology, adverse childhood experiences) [13].

This systematic review was conducted to give an updated overview of primary studies on the prevalence of mental health problems in sub-Saharan adolescents. The authors are part of an Erasmus Plus Capacity Building project focusing on youth mental health promotion in primary health care settings in South Africa and Zambia [14]. The aim was to report the outcomes of studies conveying prevalence data for depression, anxiety disorders, posttraumatic stress disorder (PTSD), emotional and behavioural problems, and suicidal behaviour in adolescents (10–19 years) across the subregion.

We elected to present a narrative synthesis of studies stratified in terms of prevalences in the general population and prevalences reported for adolescents at particular risk for mental health problems. A subsection of this review reporting outcomes of studies pertaining to HIV-infected adolescents, associates, risks, and protective factors has been published elsewhere [15]. This review might be valuable for practitioners and policy makers and also encourage further research.

## Methods

This systematic review was registered in the International Prospective Register of Systematic Reviews (PROSPERO) on 6.12.2018 (registration number CRD42018112853). The review process was guided by the Joanna Briggs Institute (JBI) Reviewer’s manual for systematic reviews of observational epidemiological studies reporting prevalence and cumulative incidence data [16].

### Search process and study eligibility

Inclusion criteria were developed and defined in accordance with the CoCoPop mnemonic (context, condition and population) recommended by JBI [16]. Study inclusion criteria were as follows:

1. Participants residing in sub-Saharan African countries as defined by the World Bank Country and Lending Groups [17].
2. Studies on adolescents between 10 and 19 years of age or a subgroup analysis for individuals within this age range. Publications were only included if separate prevalence data for adolescents with age ranging exclusively between 10–19 years of age was given.
3. English language articles published in peer-review journals.
4. Cortina et al. [13] conducted a meta-analysis of studies published until 2008. This review thus covers studies published between June 2008 and June 2020, spanning a 12-year period.
5. Studies assessing for mental disorders as well as mental health problems were included. For this review we differentiated between mental disorders and mental health problems and investigated the prevalence rates of both. We specified a mental disorder as a clinically diagnosed psychiatric condition, applying standardised classifications for mental disorders based on Diagnostic and Statistical Manual of Mental Disorders (DSM) or International Classification of Diseases (ICD) criteria. We identified mental health problems as symptoms perceived when applying a screening questionnaire, but not considered a formal mental disorder diagnosed using a standardized diagnostic instrument. Included studies used structured or semi-structured diagnostic interviews administered by trained lay workers or clinicians or applied standardised self- or caregiver/teacher-report mental health measures.
6. We included studies reporting prevalence data for depression, anxiety disorder, posttraumatic stress disorder (PTSD), emotional and behavioural problems. and suicidal behaviour (ideation, plans and attempts). For the assessment of suicidal behaviour, we also included studies that used non-standardized instruments.
7. The prevalence of a mental disorder or problems in a general population (i.e. not previously identified with mental health problems or enrolled from mental health services) was required.

**Fig 1:**
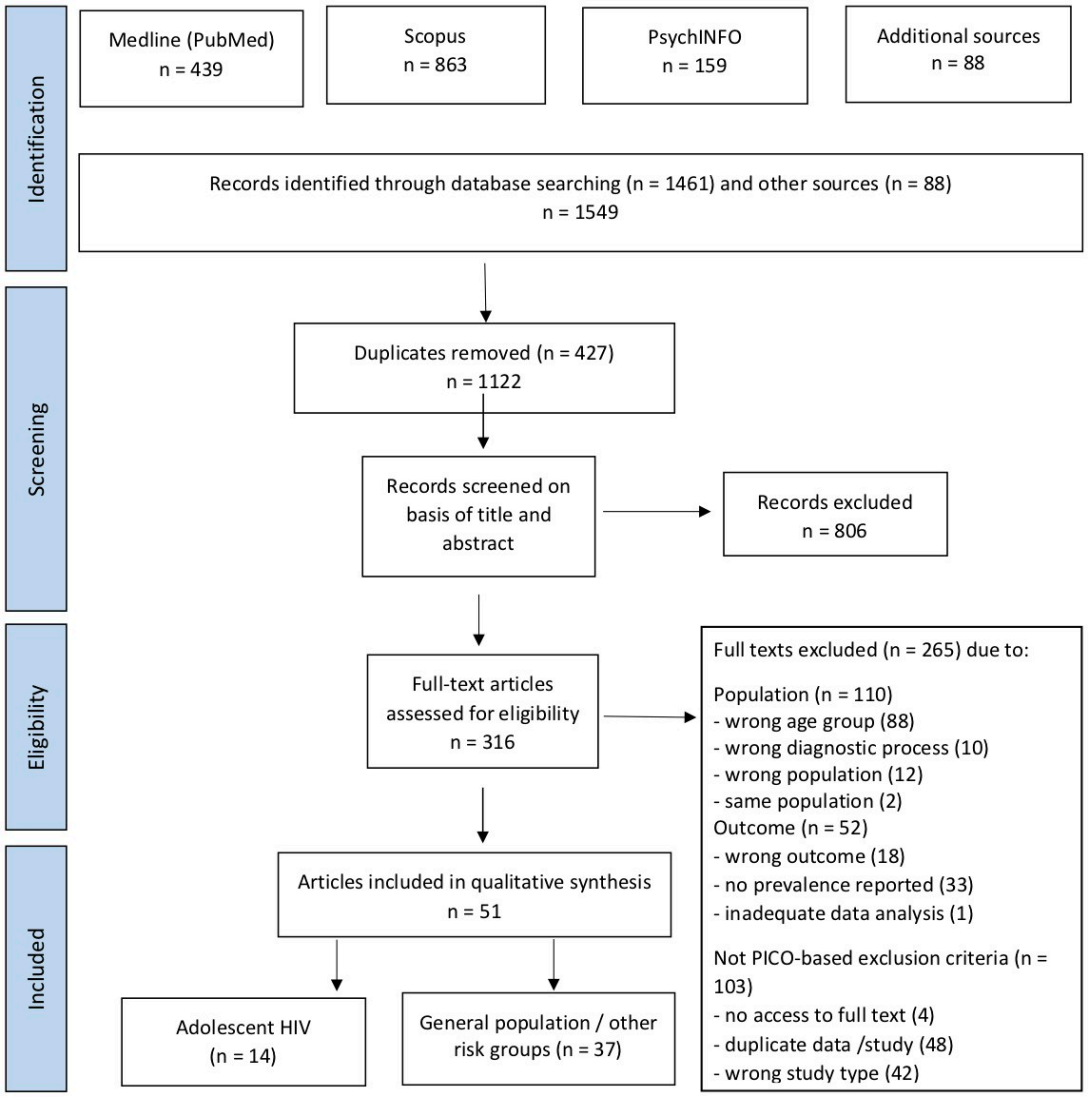
PRISMA flow chart for the systematic review process.

The databases PubMed, Scopus, and PsycINFO were searched between June and November 2018. An updated search was conducted in January 2020 to include studies published in 2019 and again in June 2020 to include articles published in 2020. The following search string was adapted for each database: child* OR youth OR adolesc* AND south africa OR zambia OR sub-saharan OR Africa AND prevalence OR incidence OR epidemiol* AND psychiat* OR mental OR depress* OR ADHD OR anxiety (full details of the search strategy are provided in the online material). An electronic search in Google Scholar and reference list checking of relevant articles were conducted to account for additional sources missed during the original search. Google Scholar and reference list of relevant articles were also searched for articles that may have been missed during the original search. Suggestions from experts in the field (e.g. authors of relevant articles, researchers from partner universities of the Erasmus project) were also sought. The Preferred Reporting Items for Systematic Reviews and Meta-Analyses (PRISMA) [18] process was followed to identify and include/ exclude the papers in this review (see Fig 1 for PRISMA flow diagram).

### Data extraction and critical appraisal

In the first step, duplicates were removed, and the remaining studies were divided between authors in Germany, South Africa and Finland with the help of the literature management program ZOTERO. Records were screened for eligibility based on title and abstract, and full text versions of eligible papers were retrieved. The following data were extracted: author, year of publication, location, country, region, sample origin, sampling method, sample size, gender ratio, assessment instrument, informant, and prevalence of psychological problems or mental disorder. Three authors independently took part in each phase of the review, data were double coded using a standardized data extraction form and disagreements were discussed to reach consensus.

Included studies were assessed for their methodological quality using the JBI Critical Appraisal Checklist [19]. The checklist consists of ten items examining representativeness of sampling, adequacy of sample size, appropriateness of study setting and recruitment, methodological quality, response rates and statistical analysis. Cultural contextualization [7] was added as a quality criterion to evaluate the extent to which authors referred to cross-cultural considerations specifically in regard to instruments used to assess mental disorders or symptoms of mental distress (full details of the quality assessment provided in the online material). A scoring system with a 10-point scale was used [20]. Studies were considered of low quality (0–4), medium quality (5–7) and high quality (8–10) in accordance with their overall score between zero and ten points. As with the data extraction, three authors reviewed studies independently and ambiguities were resolved through discussion.

### Narrative synthesis

As stated in the PROSPERO study protocol, a meta-analysis of prevalence data was originally considered for this review. However, the database search resulted in a relatively large number of eligible studies of varying quality, presenting a high degree of heterogeneity with regard to study design, outcome measures, characteristics of participant samples and statistical analysis. Given the wide range of findings, we elected not to conduct a meta-analysis, but present a narrative synthesis guided by the Joanna Briggs Institute (JBI) Reviewer’s manual for systematic reviews of observational epidemiological studies to summarize prevalence rates, instruments, and associated factors [16].

In addition, median point prevalence and interquartile ranges for depression, anxiety disorder, PTSD, emotional and behavioural problems and suicidal behaviour were reported. We defined point prevalence as prevalence measured in the past week up to six months ago (see S6 Table). Twelve-month and lifetime prevalence data were reported in an additional subsection.

## Results

Through the database search 1 549 records were identified. After the removal of 427 duplicates, abstracts and titles of 1 122 records were screened for eligibility. Twenty-two authors were contacted via email for missing prevalence data, five authors provided relevant prevalence outcomes and three of these studies were included in the review. Three hundred and sixteen studies were assessed for full text eligibility and 265 records were excluded for reasons shown in Fig 1.

**Fig 2:**
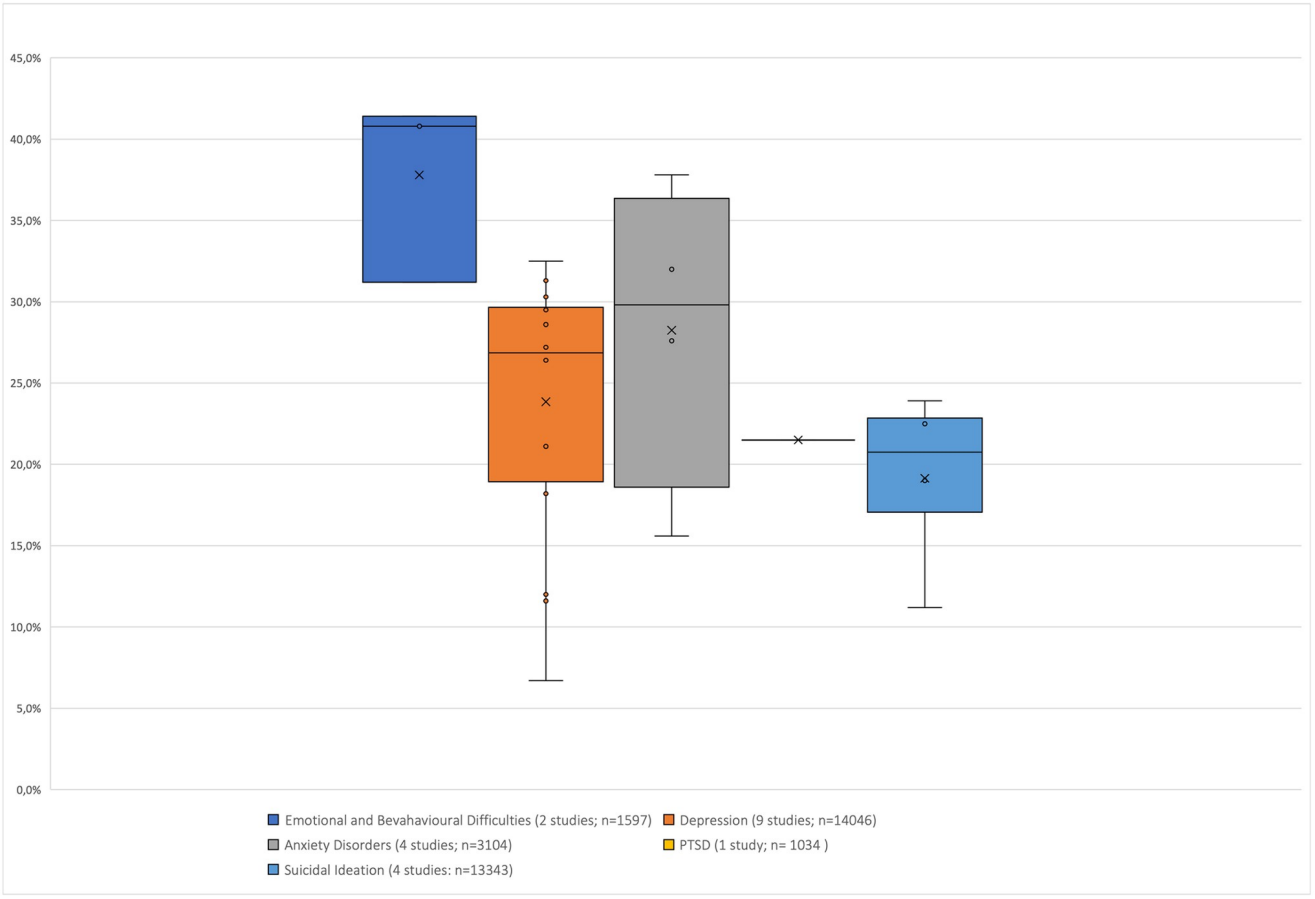
Point prevalences of adolescent mental health problems and diagnoses in the general population.

**Fig 3:**
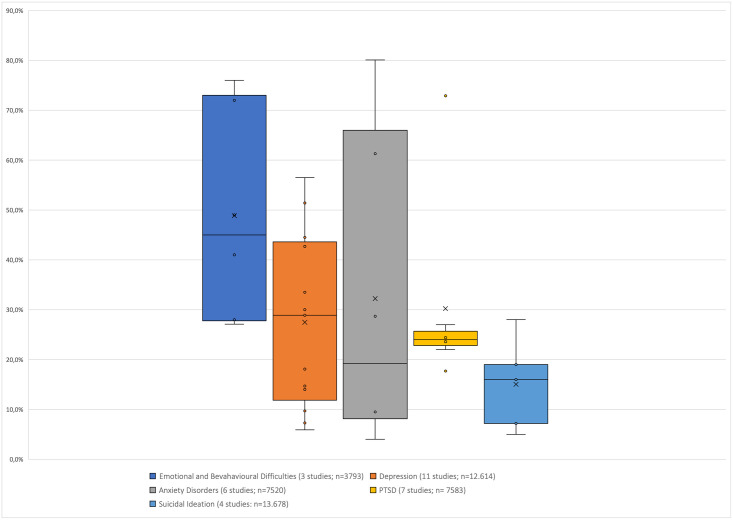
Point prevalences of adolescent mental health problems and diagnoses in at-risk groups.

Fifty-one studies met the inclusion criteria for the qualitative synthesis and were appraised for methodological quality (see S1 Table). S2 Table provides details about methodological quality. Fourteen studies focussing on HIV-positive adolescents are published elsewhere [17]. Results of 37 studies reporting prevalence outcomes of adolescent mental health morbidity as defined in this review in the general population and selected risk groups are presented in the results [21–57]. Figs 2 and 3 show the relevant median point prevalence and the interquartile ranges for the general adolescent population and at-risk groups which are described in more detail below.

### Study characteristics

Manuscripts were published between 2008 and 2020. S1 Table presents an overview of study setting, population characteristics, assessment tools and outcomes.

#### Research setting

Sixteen studies were carried out at sub-Saharan African research institutions [21, 22, 32, 33, 37–39, 41, 43, 44, 47, 50–52, 54–56] the majority in South Africa (n = 9). Nineteen of the studies were conducted in collaboration with researchers from HIC or other LMIC [13, 22–30, 35, 36, 40, 43, 48, 49, 53, 55, 57] and four studies were carried out by researchers based in HIC [34, 42, 45, 46].

Three studies [24, 46, 47] reported prevalence rates from the Global School-based Student Health Surveys (GSHS) conducted between 2003 and 2009 contributing the largest pooled data set of 31 558 adolescents from Benin, Botswana, Kenya, Malawi, Namibia, Seychelles, Tanzania, Uganda, Zambia, and Zimbabwe. Two publications [23, 43] reported prevalence rates from the African Research, Implementation Science, and Education (ARISE) Network study, based on a sample of 8 075 adolescents from nine sites in Tanzania, Ethiopia, Nigeria, Uganda, Ghana, Eswatini and Burkina Faso. Cluver et al. [29] and Cluver et al. [30] used a partially overlapping sample to report different prevalence estimates.

#### Study types

Most studies used a cross-sectional study design, four studies used baseline data from randomized control trials [35, 36, 38, 53], and two were cohort studies [30, 57]. The most common sampling method was stratified or cluster random sampling. Eleven studies reported a response rate (participants who responded to the survey/completed assessments) of ≥75% (considered adequate) [58], with six studies reporting a response rate above 90% [29, 30, 35, 38, 44, 55]. Twenty-one studies did not report a response rate. Seven studies used convenience samples and five studies used nationally representative samples [42, 43, 46, 47, 55].

#### Study setting and study population

Studies reported prevalence data from 16 different sub-Saharan countries: South Africa (19), Uganda (7), Nigeria (6), Kenya (6), Tanzania (4), Ethiopia (4), Burkina Faso (3), Ghana (3), Zambia (3), Benin (2), Botswana (2), Malawi (2), Namibia (2), Zimbabwe (2), Seychelles (1), Eswatini (1). Data were reported from 18 household samples and 19 school-based samples. Cortina et al. [31] used a school and a household sample, Cluver and Orkin [28] recruited participants from schools and community organizations and Kyohangirwe et al. [39] and Buckley et al. [25] from a primary health clinic. The majority of studies gave detailed descriptions of study sites and settings.

The total population of all studies was 97 616 adolescents, of whom 52 802 (54.0%) were female. Three studies examined female-only samples [34, 35, 38]. Sample size ranged between 81 [25] and 23 371 [24] adolescents. The mean age of participants varied between 13 and 17 years (range 10–19 years). Twenty studies were conducted among the general adolescent population, 17 studies described subgroups of adolescents exposed to one or more risk factors: poverty [26, 31, 36, 48, 56], violence and trauma [25, 27, 52], being ‘out of school’ [38], orphans and vulnerable adolescents from areas affected by HIV [28, 30, 32, 35, 40, 53, 57]. Studies were categorized according to the main risk factor.

#### Diagnostic instruments

Twenty-nine screening tools and two clinical interviews were used. A detailed overview of all measures is presented in S1 Table. Eleven of the instruments have been used previously or validated in other African settings and six studies used locally validated cut-off points in determining prevalence. The majority of studies reported on linguistic equivalence of assessment tools and conducted a pilot study or pre-test.

### Depression

Twenty studies reported point prevalence for depression in a total population of 26 660 adolescents. Twelve different screening tools and one clinical interview were used. As indicated in Fig 2, the median point prevalence for depression across nine general population studies encompassing 14 409 adolescents [21, 22, 33, 37, 39, 43, 45, 49, 50] was 26.9% and the interquartile range (IQR) was 20.1–31.1 The median point prevalence for 11 studies (n = 12 614) with at-risk samples [25–28, 30, 35, 36, 38, 48, 52, 53] was 29.0% (IQR 12.9–42.0) as shown in Fig 3.

There was only one study that assessed lifetime prevalence of depression. Ward et al. [55] used a household sample of 5 631 South African adolescents (aged 15–17) and reported a lifetime prevalence of 2.6% (m: 3.1% vs. f: 2.0%) for symptoms indicative of depression using the Trauma Symptom Checklist for Young Children.

#### Prevalence of depression in the general population

Two out of nine studies on depression prevalence among the general adolescent population used a diagnostic interview, seven used screening instruments. The two studies that used a clinical interview were both conducted in Uganda, one in households of disadvantaged districts [37], the other one in primary care [39]. Kinyanda et al. [36]. measured depression with the Mini International Neuropsychiatric Interview (MINI Kid) in a community sample of 1 587 children and adolescents aged 13–19 years from four disadvantaged districts (Lira, Tororo, Kaberamaido, and Gulu) in rural North-Eastern Uganda. For a subsample of 897 adolescents (aged 10–19) the reported prevalence of at least one DSM-IV psychiatric diagnosis was 38.5%, with 11.6% fulfilling the criteria for a depressive disorder (major depressive episode, dysthymia). Kyohangirwe et al. [38] conducted diagnostic interviews (MINI Kid) among a primary care convenience sample of 281 adolescents (63.0% females) aged 10–17 in Kampala, Uganda. The prevalence of major depressive disorder was 18.2%. Younger adolescents (aged 10–13) reported a lower prevalence than older adolescents (aged 14–17) (11.9% vs. 21.7%). Vulnerability factors reported were exposure to childhood trauma and orphanhood.

The other seven studies used various screening instruments among school samples. Nyundo et al. [42] reported on the prevalence of depressive symptoms from household samples (n = 7 662) in six Sub-Saharan countries (Burkina Faso, Ethiopia, Ghana, Nigeria, Tanzania and Uganda) using the Kutcher Adolescent Depression Scale-6 (KADS-6). Data were reported for five urban and four rural subsample populations (n = 7 662) of both in-school and out-of-school adolescents (mean age 14.4). The highest prevalence of symptoms of depression was 32.5% for adolescents from rural Ghana (n = 625). Ethiopian adolescents living in urban Harar (n = 1 059) had a higher prevalence (28.8%) compared to a rural sample from Kersa (n = 951; 21.1%). In Tanzania, the prevalence rate in urban (n = 1 226) and rural (n = 825) sites was comparable (Dar es Salaam: 31.3%, Dodoma: 30.3%). The prevalence of adolescents from Ibadan, Nigeria (n = 750) was 27.2% and similar levels of depressive symptoms were found in population groups in rural areas in Uganda (Iganga/Mayuge, n = 598; 26.5%) and Burkina Faso (Nouna, n = 1 628; 26.4%).

Adeniyi et al. [21] administered the Children Depression Inventory (CDI) to 1 100 students (mean age 15.2) from public and private secondary schools in Ibadan, Nigeria. The reported prevalence was 23.8% for mild to moderate depressive symptoms and 5.7% met severity of depression. Depressive symptomatology was associated with a low level of physical activity and was more prevalent among females and adolescents of older age.

Another study employed the Patient Health Questionnaire-9 (PHQ-9) among 1 713 adolescents (mean age 14) attending four private and three public secondary schools in Southwest Nigeria [33]. The depression prevalence was 21.2%, with 16.1% screening positive for moderate, 4.6% for moderately severe, and 0.5% for severe depression symptoms. Factors associated with symptom severity were not living with parents, a large number of siblings, a recent change in residency and a low level of physical activity.

Osborn et al. [45] used the Patient Health Questionnaire-Modified for Adolescents-8 (PHQ-8) in a convenience sample of 658 high school students (mean age 15.8) in Nairobi, Kenya. The overall prevalence was 45.90%, with 35.0% reporting mild, 29.2% moderate, and 16.9% severe depressive symptoms. Depressive symptoms were significantly associated with older age.

Three studies reported prevalence rates from South African school samples. Stansfeld et al. [49] administered the Short Moods and Feelings Questionnaire (SMFQ) to 1 034 Grade 8 learners (mean age 14.2) from Cape Town and reported a 41.2% prevalence of clinically significant depressive symptoms, with more females than males scoring positive. The sample was characterized by high levels of exposure to violence in the previous year (84.1%), with those experiencing the highest levels of violence showing the highest rates of depressive symptoms.

Bach and Louw [22] administered the CDI to a sample of 377 Venda and Northern Sotho high school students (aged 15–18) in the rural Soutpansberg district in the Limpopo Province. Witnessing or experiencing violence was associated with depressive symptomatology in both ethnic groups. The prevalence of depressive symptoms was higher among females (Sotho: 28.6%, Venda: 29.7%), compared to males (Sotho: 12.1%, Venda: 6.7%).

In comparison, Strydom et al. [50] assessed 515 secondary school students (aged 16–18 years) from four public schools in Bloemfontein and reported a prevalence of 19.7%, with 14.0% presenting mild and 5.3% showing moderate to severe symptoms on the depression subscale of the Hospital Anxiety and Depression Scale (HADS). The prevalence among older students was lower (15.9%) than for younger students (23.8%).

#### Prevalence of depression in at-risk populations

*Adolescents living in poverty*. The Study of the Well-being of Adolescents in Vulnerable Environments (WAVE) [26] used the 10-item version of the Center for Epidemiologic Studies Depression Scale (CES-D) to investigate the prevalence of depression among adolescents from deprived neighbourhoods in Baltimore, New Delhi, Ibadan, Johannesburg, and Shanghai. The highest prevalence for depressive symptoms was reported for a subsample of 496 adolescents (aged 15–19) from Johannesburg, South Africa, with 44.6% of females and 41.1% of males scoring above the cut off, followed by male (29.4%) and female (28.5%) adolescents from Ibadan, Nigeria (n = 449). A significant gender difference in depression symptoms was found for adolescents from Johannesburg, but not for Ibadan. Across countries school enrolment rates were the lowest among males from Ibadan (15.8%) and males from Johannesburg (22.9%).

Shangani et al. [48] reported baseline data from the Orphan and Separated Children’s Assessments Related to their Health and Well-Being (OSCAR) study. The rural sample of 655 adolescents (mean age 14) was obtained from eight sites in Uasin Gishu county, located in western Kenya. High rates of reported food insecurity (78.8%), exposure to sexual abuse (12.5%) and transactional sex (19.2%) characterized the sample, with 56.5% of the adolescents presenting scores on the short version of the CDI indicative of depression [47].

In a sample of 360 ultra-poor young female adolescents (mean age 12.6) and their mothers, derived from twelve rural sites in the Nord region of Burkina Faso, the reported prevalence of depressive symptoms was 14.7% using the 20-item CES-D [36]. More females (15.7%) scored above the cut-off than males (13.9%) [35]. Trauma and depression were most prevalent among adolescents reporting high levels of exposure to violence, adolescents who were not enrolled in school and adolescents engaged in exploitative child labour.

*Out-of-school adolescents*. Among out-of-school adolescent girls and young women (AGYW) in North-West Tanzania (n = 1 322) a depression prevalence of 30.0% was reported using the PHQ-2 [38]. Living alone or with younger siblings and having experienced violence from a sexual partner were associated with depressive symptoms.

*Adolescents who experienced trauma*. Suliman et al. [51] reported prevalence data for a subsample of 922 Grade 10 high school students (59% females; mean age 15.7) from Cape Town, who reported DSM-IV qualifying trauma exposure. The prevalence of severe symptoms of depression measured on the Beck Depression Inventory (BDI) was 7.3%, with females scoring significantly higher than males. Exposure to multiple severe traumatic events increased the level of depressive symptoms.

Closson et al. [27] reported for a sample of 767 adolescents (mean age 17) from Soweto a prevalence of 33.5% for probable depression using the 20-item CES-D. A higher prevalence was reported for females (36.4%) than males (29.5%) and depression prevalence was associated with a high potentially traumatic event (PTE) score. Between 40% and 70% of all adolescents experienced at least one violent event in the community, and nearly half of the adolescents experienced seven PTEs measured with the Traumatic Event Screening Inventory-Children (TESI-C).

Buckley et al. reported a prevalence rate of 14.0% for depression using the PHQ-A in a community sample of 81 HIV-uninfected adolescents (aged 13 to 19 years; 65% female) from Soweto [25]. The prevalence was 5.0% for minor depressive disorder, 7.0% for dysthymic depressive disorder, and 7.0% scored above the cut-off for major depressive disorder. Associated factors reported for depression were experiencing aggression or anger problems and witnessing violence at home. The study showed rates of exposure to violence similar to Closson et al. [27], with more than 50% reporting they had witnessed violence in the community and 69% that they had experienced more than one PTE.

*Orphans and vulnerable adolescents*. Four studies reported a depression prevalence among orphans and vulnerable adolescents from HIV endemic regions in South Africa. Cluver and Orkin [28] administered the CDI to 1025 adolescents (aged 10–19) from deprived Xhosa-speaking neighbourhoods in Cape Town, including street children, adolescents from child-headed households, adolescents not attending school, AIDS-and other cause orphans. Clinical-level depression prevalence was 9.7%. The sample was assessed for depression, anxiety and PTSD, with 31% of the adolescents experiencing at least one disorder. AIDS-orphans were more likely to score above cut-off for any disorder. Food insecurity, stigma and bullying each independently increased the likelihood of mental health problems. In combination, poverty and stigma increased the odds for any internalizing disorder from 19% to 83% and AIDS-orphanhood and bullying from 12% to 76%.

Goin et al. [35] used a baseline sample of 2 533 AGYW from a randomized trial of a cash transfer conditional on school attendance to investigate mediating factors in the relationship between depressive symptoms and HIV incidence in rural Mpumalanga Province. The study reported a prevalence of 18.1% for female adolescents aged 13 to 19 using the 10-item CDI and confirmed an increased risk of cumulative HIV incidence among depressed adolescents. Cluver et al. [30] reported a depression prevalence of 5.9% (m: 4.5% vs. f: 7.3%) using the 10-item CDI in a sample of 3 515 South African adolescents (aged 10–18) from Mpumalanga and the Western Cape.

Thurman et al. [53] investigated 489 adolescents (mean age 15.5) enrolled in a support program for HIV-affected and vulnerable families in two rural districts of the Eastern Cape Province. At baseline, significant depressive symptoms were reported by 51.5% of adolescents in the intervention group and 44.5% in the control group.

### Anxiety

Ten studies reported point prevalence outcomes for anxiety disorders and symptoms in a total population of 10 624 adolescents using eight different self-report questionnaires and one diagnostic tool. The median point prevalence of four general population studies (n = 3 104) was 29.8% (IQR 18.6–36.4) [37, 45, 49, 50]. In the six at-risk studies (n = 7 520) the median point prevalence was 19.3% (8.1–66.0) [25, 28, 30, 38, 48, 52].

The only study assessing lifetime prevalence of symptoms indicative of anxiety disorders reported a prevalence of 3.4% (m: 3.2% vs. f: 3.6%) for a sample of 5 631 South African adolescents (aged 15–17) using the TSCYC [55].

#### Prevalence of anxiety in the general population

Three of the studies were conducted in schools [45, 49, 50], one in households [37]. Stansfeld et al. reported a prevalence of 15.6% using the 19-item Zung Self-Rating Anxiety Scale (SAS) in a sample of Grade 8 learners from Cape Town, South Africa (n = 1 034, mean age 14.2) [49]. Participants reporting the highest levels of exposure to violence were more likely to suffer from anxiety. Strydom et al. screened for anxiety in 515 adolescents (aged 16–18 years) from schools in Bloemfontein, South Africa and found an overall prevalence of 61.2%, with 29.0% reporting mild and 32.0% reporting moderate to severe symptoms on the anxiety subscale of the HADS [50]. In a sample of Kenyan high school students (n = 658; mean age 15.8) from Nairobi anxiety was assessed with the Generalized Anxiety Disorder Screener-7 (GAD-7) with a reported prevalence of 35.7% for mild anxiety, 25.5% for moderate anxiety, and 12.3% for severe anxiety [44]. Female adolescents, older adolescents and members of a minority tribe presented with higher scores for anxiety. Kinyanda et al. [36] used a diagnostic measure (MINI Kid) and reported a prevalence of 27.6% for anxiety disorder syndromes in a household sample of 897 adolescents aged 10–19 from rural Uganda.

#### Prevalence of anxiety in at-risk populations

*Adolescents living in poverty*. Shangani et al. [48] administered the Children’s Manifest Anxiety Scale—Revised (R-CMAS) among orphaned and vulnerable Kenyan adolescents (n = 655, mean age 14) and reported the highest prevalence of 80.1%.

*Out-of-school adolescents*. The Generalized Anxiety Disorder Screener– 2 (GAD-2) was administered among a Tanzanian community sample of 1 322 adolescent girls (aged 15–19) not enrolled in school and a prevalence of 28.7% was reported [38]. Exposure to violence from a sexual partner and HIV-positive status were associated with anxiety symptoms.

*Adolescents who experienced trauma*. Suliman et al. administered the Multidimensional Anxiety Scale for Children (MASC) among 922 Grade 10 students (58.8% females; mean age 15.7) from Cape Town who had experienced multiple life-threatening traumatic events and reported a prevalence of 61.3%, with female adolescents reporting more anxiety symptoms [52]. Buckley et al. [25] administered the PHQ-A to a sample of 81 HIV-uninfected adolescents (aged 13–19; 65% female) from Soweto, South Africa, and reported an overall prevalence of 4.0% for anxiety disorder.

*Orphans and vulnerable adolescents*. Cluver and Orkin [28] used the R-CMAS to estimate anxiety among 944 orphaned and vulnerable children and adolescents (aged 10–19) from poor urban Xhosa-speaking neighbourhoods of Cape Town, South Africa and reported a prevalence of 9.8%. Cluver et al. [30] used the R-CMAS in a sample of 3 515 South African adolescents from Mpumalanga and the Western Cape (aged 10–18) and reported a prevalence of 9.5% (m: 7.5% vs. f: 11.5%).

### Emotional and behavioural problems

Five studies with a total population of 5 390 adolescents reported on emotional and behavioural problems using the Strengths and Difficulty Questionnaire (SDQ). The median point prevalence in two general population studies (n = 1 597) was 40.8% (IQR 31.2–41.4) [37, 42]. The median point prevalence in three at-risk group samples (n = 3 793) was 45.0% (IQR 27.8–73.0) [31, 32, 40].

#### Prevalence of emotional and behavioural problems in the general population

One of the studies was conducted in a secondary school, the other in the community. In a nationally representative sample of 700 Tanzanian secondary school students (mean age 14.9), parent-reported rates of emotional and behavioural problems were lower (31.2%) in comparison to self-reported outcomes (40.8%) [42]. Parents also estimated a lower prevalence of peer problems (53.8% vs. 63.0%), conduct problems (34.5% vs. 45.0%), emotional symptoms (36.6% vs. 39.6%) and hyperactivity (16.5% vs. 16.7%). The study found a strong association between adolescent mental health problems and exposure to physical violence by their parents or caregivers. An equally high self-reported prevalence (41.4%) was found by Kinyanda et al. [36] in community sample of 897 adolescents (aged 10–19) from four rural districts in Northeastern Uganda.

#### Prevalence of emotional and behavioural problems in at-risk populations

*Adolescents living in poverty*. In a rural South African school-based sample of 1 025 learners (aged 10–12), teachers identified elevated rates of overall emotional and behavioural problems (41.0%) and difficulties with social behaviours (15.2%), while self-reported scores on the Youth Self Report (YSR) indicated lower, but significant rates (14.1%) of anxiety and depression [31]. Mother’s partnership status, mother’s education level, and second-generation refugee status were found to aggravate the occurrence and severity of mental health problems.

*Orphans and vulnerable adolescents*. Lachman et al. [40] used the parent-report version of the SDQ in a large community sample of 2 477 caregiver-child dyads obtained from areas of KwaZulu-Natal, South Africa with a high HIV prevalence. The stratified sample included 27.7% dyads with an AIDS-ill caregiver and 7.4% dyads with AIDS-orphans. The overall prevalence of emotional and behavioural problems was 13.5% in the borderline range and 13.6% in the clinical range, with HIV-affected families reporting more child behaviour problems.

The highest self-reported prevalence of emotional and behavioural problems was found in a sample of 291 young mostly school-going adolescents (mean age 13) affected by HIV/AIDS in Ghana [32]. The criteria for overall psychological distress were fulfilled by 76.0% of orphaned and vulnerable children, 72.0% of children with HIV-infected parent/caregiver, 49.0% of other-orphans and 28.0% of non-orphaned and vulnerable children. A comparison of the self-reported results versus caregiver-reported results for the different SDQ subcategories, revealed a low inter-informant agreement, with caregivers reporting a higher score of total difficulties and more conduct problems, whereas adolescents reported more emotional and peer-related problems.

### Posttraumatic stress disorder

Eight studies reported on the point prevalence for PTSD for a total population of 8 617 adolescents. Four self-report measures, and one clinical interview measure were used. Stansfeld et al. presented the only general population study. The median point prevalence for seven at-risk-group studies (n = 7 583) was 24% (IQR 22.0–27.0) [25, 28, 30, 35, 47, 51, 56].

Ward et al. [55] assessed the lifetime prevalence of PTSD among a household sample of 5 631 South African adolescents (aged 15–17) using the TSCYC and reported a prevalence of 1.6% (m: 2.1% vs. f: 1.0%) for PTSD. Sexual violence was found to be strongly associated with mental health conditions.

#### Prevalence of posttraumatic stress disorder in the general population

Stansfeld et al. [48] used the Harvard Trauma Questionnaire (HTQ) in a sample of 1 034 Grade 8 students (mean age 14.2) from Cape Town, South Africa and reported a prevalence for PTSD of 21.5%. The study found that those falling in the highest quartile of violence were most likely to score positive for symptoms of PTSD.

#### Prevalence of posttraumatic stress disorder in at-risk populations

*Adolescents living in poverty*. Shangani et al. [48] evaluated posttraumatic stress symptoms using the Child PTSD Checklist in a sample of 655 Kenyan adolescents (mean age 14) living in extremely poor households and reported a prevalence of 72.9%, with older adolescents showing higher rates of psychopathology. Cortina et al. [31] administered the Trauma Symptom Checklist for Children—Alternate form (TSCC-A) to a rural South African school sample of 1 025 socially disadvantaged young adolescents (aged 10–12) and reported a prevalence of 24.0% for posttraumatic stress symptoms which were associated with older age.

In a sample of 360 adolescents (mean age 12.6) from 8 ultra-poor villages in the Nord region of Burkina Faso [36], the Children’s Revised Impact of Events Scale-8 (CRIES-8) was administered and 17.8% (m: 18.56 vs. f: 16.87) scored above the cut-off. Children experiencing violence at home, at work and in the community were more likely to experience depression and trauma.

*Adolescents who experienced trauma*. Suliman et al. [51] assessed 922 trauma-exposed high school students (58.8% females; mean age 15.7) from Cape Town with the Child PTSD Checklist and reported a prevalence of 23.6%, with higher rates of PTSD reported for females. Most of the adolescents were exposed to ≥ 1 (and up to 6) traumatic events (75.3%) and higher rates of trauma exposure was associated with a higher occurrence of PTSD [51]. In a sample of 81 HIV-uninfected adolescents (aged 13–19; 65% female) from Soweto, South Africa, the prevalence of PTSD symptoms was 22.0% using the PHQ-A [25].

*Orphans and vulnerable adolescents*. In a sample of 1 025 vulnerable and orphaned adolescents (aged 10–19) the prevalence of PTSD was 27.0% and an association between poor care and any disorder (PTSD, anxiety disorder or depression) was reported [28]. In a sample of 3 515 South African adolescents from Mpumalanga and the Western Cape (aged 10–18) the prevalence of PTSD 21.7% for male and 27.6% for female adolescents Both studies conducted diagnostic interviews using the Child PTSD Checklist [30].

### Suicidal behaviour

Eight studies reported point prevalence estimates for suicidal behaviour (ideas, plans, and attempts) for a total of 27 021 adolescents. The majority of studies measured suicidal behaviour as a dichotomous variable using single questions and two studies used a diagnostic measure. The median point prevalence of suicidal ideation for four general population studies (n = 13 343) was 20.8% (IQR 13.2–23.6) [34, 41, 51, 56] and across the four at-risk samples (n = 13 678) the median point prevalence of suicidal ideation was 11.6% (IQR 5.6–25.0) [25, 29, 30, 57].

#### Point-prevalence of suicidal behaviour in the general population

A point prevalence was reported from three school samples [41, 51, 56] and one community survey [34]. In the 2008 South African Youth and Risk Behaviour Survey (YRBS), an adapted version of the Youth Risk Behaviour Surveillance System (YRBSS) of the US Centers for Disease Control was administered to a representative sample of 10 270 high school students (mean age 16.1), with two questions inquiring about suicidal behaviour [51]. The survey revealed a prevalence of 19.0% for suicidal ideation and 21.8% for attempting suicide at least once in the last six months, with females reporting suicidal ideation (21.4% vs. 17.3%) and attempts (22.7% vs. 20.8%) more frequently than males. Female sex, older age, feelings of hopelessness, feeling unsafe, having unsafe sex, body dissatisfaction, exposure to violence and substance abuse were associated with suicidality.

Vawda et al. [56] assessed suicidal behaviour among a convenience sample of 222 Grade 8 learners (mean age 13.3) from a middle school located in a resource-constrained setting in Durban. Reported prevalence was 22.5% for suicidal ideation, 5.9% for suicide plans and 5.4% for suicide attempts. High scores on the BDI and the Perceived Stress Scale (PSS), suicidal ideation expressed by peers, and alcohol use were strong predictors for suicidal behaviour.

Mashego and Madu [41] reported the prevalence of suicide-related behaviours among an ethnically diverse sample of mostly female (60.6%) secondary school students (n = 142; mean age 16.2) in the Welkom and Bethlehem areas of the Free State using an eight-item scale and a single question. Of the whole sample 23.9% (m: 26.8% vs. f: 22.1%) reported suicidal ideation often or almost every day and 12.0% (m: 10.7% vs. f: 12.8%) at least almost every day in the past two weeks. Suicidal plans in the past two weeks were reported by 7.7% (m: 5.4% vs. f: 9.3%) and suicidal attempts by 4.2% (m: 0.0% vs. f: 7.0%). No significant gender, age or ethnic group differences were found.

Gage et al. [34] measured suicidality with two binary items in an all-female household sample of 2 079 adolescent girls (mean age 14.2) obtained from a survey of early marriage prevention in the Amhara Region in northern Ethiopia. The study reported a prevalence of 11.2% for suicidal ideation and 2.3% for suicide attempts in the last three months, with the 5.2% who were ever married and the 5.4% who were promised in marriage having higher odds for reporting suicidal ideation and behaviour.

#### Point-prevalence of suicidal behaviour in at-risk populations

*Adolescents who experienced trauma*. In a sample of 81 HIV-uninfected adolescents (aged 13–19; 65% female) from Soweto, South Africa, a prevalence of 28% for suicidal ideation was reported using the PHQ-A [25]. Female sex, repeating a grade in school and experiencing sexual or physical abuse or rape were associated with a higher likelihood of suicidality.

*Orphans and vulnerable adolescents*. In a large community survey, 5 998 adolescents (mean age 13.5) completed the suicidality subscale of the MINI Kid [29]. The sample was drawn from three districts with over 30% antenatal HIV prevalence and 25% of the participants were HIV-affected (Western Cape, Mpumalanga, and KwaZulu-Natal). Suicidality prevalence was 5.0%, with higher rates reported for females compared to males (6.5% vs. 3.2%), for older adolescents compared to younger adolescents (7.6% vs. 2.6%; median split 13 years), for children orphaned by AIDS compared to children who were not orphaned by AIDS (8.1% vs. 4.6%) and for children with an AIDS-ill caregiver compared to children without an AIDS-ill caregiver (7.1% vs. 4.3%) [29].

For a subset of the same sample (n = 3515; aged 10–18 years; 56% females) from Mpumalanga and the Western Cape, prevalence rates for suicidal ideation, suicide plans, and suicide attempts were 7.2%, 5.8%, and 3.2% [30]. In this prospective study by Cluver et al. showed that cumulative adverse childhood experiences (ACE), such as orphanhood by AIDS and parental AIDS-related illness, food insecurity and parental abuse, predicted suicidality rates at follow-up one year later, from 1.9% (no ACE) to 6.3% (>5 ACE). Depression, anxiety and posttraumatic stress had a mediating effect on the association.

Zietz et al. [57] examined suicidal ideation in a sample of 4 084 adolescents from the HIV-endemic Nyanza region of western Kenya. Data was derived from the baseline data of an observational cohort study evaluating the effects of HIV testing and disclosure on adolescent behaviour and well-being, A large proportion of the sample were orphans (41.0%) but only 17 participants were HIV-infected due to the exclusion criteria of the original research study. The prevalence of suicidal ideation was 16.0% using questions from the CESD-R, of which 12.0% were considered to be at moderate to high-risk of suicide attempt. Female sex, being sexually active, impregnating a partner (in males) and experiencing abuse increased the likelihood of suicidal ideation.

#### Twelve-months prevalence and lifetime prevalence of suicidal behaviour

Twelve-months prevalence rates for suicidal behaviour were reported by nine studies [23, 24, 26, 37, 43, 44, 46, 47, 54] and lifetime prevalence by three studies [26, 37, 41].

#### Twelve-months prevalence and lifetime prevalence in general population studies

Seven studies were conducted in schools [24, 41, 44, 46, 47, 54] and three in households [23, 37, 43]. Two studies reported a 12-month prevalence for suicidality among national representative school samples [46, 47] of overall 29 418 adolescents (aged 13–15) from nine sub-Saharan countries that participated in the Global School-based Student Health Surveys [GSHS] between 2003 and 2009. Twelve-month prevalence for suicidal ideation ranged from 11.6% (m: 10.1% vs. f: 12.7%) reported from Malawi to 31.1% (m: 31.0% vs. f: 30.8%) reported from adolescents in Zambia. Total12-month prevalence for suicide plans among adolescents from Kenya, Namibia, Uganda, Zambia and Zimbabwe was 29.3% (m: 28.5% vs. f: 29.9%), with Kenyan adolescents reporting the highest (39.6%) and Ugandan adolescents the lowest prevalence (20.8%). Brown et al. [24] pooled GSHS data collected between 2003 and 2006 from 20 896 adolescents from Kenya, Namibia, Tanzania, Uganda, Zambia and Zimbabwe and reported a total 12-months prevalence of 20.0% for suicidal ideation and 22.0% for suicide plans. The study reported an association between days bullied and suicidal ideation and plans.

Omigobodun et al. [44] measured suicidality using two items of the Disc Predictive Scales among 1 429 adolescents (mean age 14.4) attending secondary schools in five urban and six rural districts in Ibadan and the surrounding area of southwest Nigeria. The 12-month prevalence was 22.9% for suicidal ideation and 11.7% reported having attempted suicide in the past year. The study showed a significant correlation between suicidal ideation and sexual abuse, exposure to physical violence, working to support the family, hunger, parental divorce and living in a polygamous family. Exposure to violence, sexual abuse and living in urban settings increased the odds for suicide attempts [43]. In comparions, 12-month prevalence of suicidal ideation and behavior was 32.2% (m: 25.6% vs. f: 38.6%) among a convenience sample (n = 87) of Grade 9 and 10 adolescents (mean age 15.2) attending a private high school in Cape Town [54].

Among 142 high school students (mean age 16; 60.6% females) from the Free State the lifetime prevalence for suicide plans was 18.3% (m: 14.3% vs. f: 20.9%) and 14.8% (m: 12.5% vs. f: 16.3%) reported ever having attempted suicide [41].

Two studies [23, 43] reported results from the ARISE Network Adolescent Health Study, a household study that surveyed 8 075 adolescents (mean age 14.4) in households from nine sites in Tanzania, Ethiopia, Nigeria, Uganda, Ghana, Eswatini and Burkina Faso with three questions from the Global School Health Questionnaire (GSHQ). A 12-months prevalence of 4% for suicidality (ideation, plans, and attempts) in the total population was reported, with adolescents from Ibadan, Nigeria showing the highest overall prevalence rates for suicidal ideation/behaviour (12.4%), suicide plans (5.9%) and suicide attempts (6.4%). Females more frequently reported suicidal ideation/behaviour and plans across all sites, except Kersa, Ethiopia and Nouna, Burkina Faso. Suicidal ideation and behaviours were associated with female sex, older age, feeling lonely, worries that lead to sleeplessness, poor access to health care, bullying and food insecurity. Adolescents were four times more likely to report suicidal ideation if they had experienced depressive symptoms or had experienced violence in Harar and Ibadan.

One study reported lifetime prevalence for suicidality in a community study and four studies used school samples. Kinyanda et al. measured lifetime adolescent suicidality in a rural community sample of 897 adolescents from Uganda using three questions in the MINI Kid inquiring about suicidal thoughts, deliberate self-harm and suicide attempts. Lifetime prevalence of suicidal ideation was 6.1%, with a lower prevalence rate reported for male (4.0%) than for female adolescents (8.0%). Lifetime prevalence of deliberate self-harm was 1.4% and 1.7% had ever attempted suicide. Suicidality was most prevalent in adolescents living in Kaberamaido, a district not affected by war or the most severe poverty. Associated factors reported were district of residence, female sex, older age, lower level of education and income, not living with parents, orphanhood, exposure to war and a psychiatric diagnosis and mental distress.

#### Twelve-months prevalence and lifetime prevalence of suicidal behaviour in at-risk populations

*Adolescents living in poverty*. The WAVE study [26] reported a 12-months prevalence for suicide plans and attempts among adolescents from low-socioeconomic areas in Baltimore, New Delhi, Ibadan, Johannesburg, and Shanghai using three binary items. The highest percentage for suicide attempts in the last 12 months was reported for female (14.3%) and male (18.3%) adolescents from Ibadan, Nigeria (n = 449; aged 15–19). Females who did not have a caring adult and no supportive neighborhood were more likely to develop suicidal ideation, while peer support was reported to be protective for female and male adolescents. Lifetime prevalence for suicidal thoughts and suicide plans was the highest in Johannesburg, South Africa (n = 496; aged 15–19) among all subsamples, with 39.6% of females and 32.6% of male adolescents reporting having had suicidal thoughts and 25.0% of females and 19.9% of males having made plans to commit suicide.

## Discussion

This systematic review highlights the magnitude of mental disorders and mental health symptoms among adolescents in the general population and at-risk adolescents living in sub-Saharan Africa. Across 37 studies, published since 2008, spanning 97 616 adolescents, we found the following median point prevalences in the general population: 26.9% (IQR 20.1–31.1) for depression, 29.8% (18.6–36.65) for anxiety disorders, 40.8% (IQR 31.2–41.4) for emotional and behavioural problems, 21.5% in our only study for PTSD, and 20.8% (IQR 13.2–23.6) for suicidal ideation.

We also reviewed studies of at-risk adolescents encompassing adolescents affected by HIV and AIDS, exposure to violence and trauma, poverty, orphanhood, being ‘out of school’, socioeconomic disadvantages and high levels of deprivation. The point prevalences in at-risk samples were as follows: 29.0% (IQR 12.9–42.0) for depression, 19.3% (8.1–66.0) for anxiety disorders, 45.0% (IQR 27.8–73.0) for emotional and behavioural problems, 24% (IQR 22.0–27.0) for PTSD, and 11.6% (IQR 5.6–25.0) for suicidal ideation/suicidality.

A number of studies reported higher prevalence rates for adolescents orphaned by AIDS and living with AIDS-ill parents, which is in line with evidence showing an association between parental physical or mental illness and increased child and adolescent mental health morbidity [59]. In the separately published subsection of our systematic review [17] median point prevalences for 14 studies on sub-Saharan adolescents living with HIV were as follows: 22.2% (IQR 15,5–41,1) for depression, 25.0% (IQR 14.7–45.6) for anxiety, 28.5% (IQR 9–29.1) for emotional and behavioural problems, 5% for PTSD (one study), and 17.6% (IQR 9.3–23.3) for suicidal ideation/suicidality.

A detailed discussion of associated factors reported in the studies is beyond the scope of this article, for an overview of the above-mentioned factors see S3 Table. Most frequently reported in the general population and at-risk groups were female sex, older age and exposure to different forms of violence and traumatic events. These findings are consistent with systematic reviews from both, sub-Saharan and other low-and middle-income countries [13, 60–62]. This review focused on data from only 16 of the 48 countries of sub-Saharan Africa. There is a therefore a great need to gain a better understanding of the many different mental health challenges adolescents across sub-Saharan Africa face and to reflect on the possible protective factors [63].

Furthermore, a number of studies have been conducted in conflict/post-conflict settings (e.g. South Africa, Uganda, Namibia, Nigeria). Damage to vital infrastructure, including schools and health services, had a profound impact on physical and psychological wellbeing of populations affected by civil wars/conflicts. In youth, mental health problems, functional impairment and psychosocial adjustment have been shown to be not only impacted by exposure to traumatic events sustained as a result of the conflict, but also by post-conflict school attendance, community acceptance, family support and economic opportunities. A pooled rate comparison of conflict versus non-conflict settings was presented in a recent systematic review and meta-analysis of the prevalence of PTSD from studies from sub-Saharan Africa [64]. We suggest that future systematic reviews of adolescent mental health problems in sub-Saharan Africa also present results stratified in terms of conflict/post-conflict countries.

Overall, the prevalence rates reported for sub-Saharan adolescents in this review are high in comparison with studies from European countries and the USA [60–62] and other reviews of adolescent mental health problems in resource-constrained settings [5, 7, 13, 63–65]. However, reviews by Cortina et al. [13] and Kieling et al. [5] included younger participants aged 0 to 16 years, whereas this review focused on adolescence, a typical age of onset and manifestation of mental health disorders [66, 67].

The high prevalence rates reported in this review should be viewed with caution for two reasons. Female adolescents were overrepresented (54.0%) in the total population of all studies included. Globally, females are more frequently exposed to risk factors, such as gender discrimination, exposure to violence and sexual abuse, and more frequently affected by internalizing mental health disorders [65, 66]. Furthermore, the majority of studies included utilised screening tools, which have been shown to overestimate prevalence rates compared to diagnostic interviews [13, 67].

This review indicates that there has been an increase in the number of epidemiological studies examining child and adolescent mental health in sub-Saharan Africa over time being, and that the majority fulfil high quality standards with regard to research methods. Research directions that deserve further emphasis include the need for longitudinal studies, the need for more work on risk and resilience factors, and the need to integrate public health and neuroscience perspectives [68]. The critical appraisal of the included studies underlines the need for locally validated tools and a focus on cross-cultural contextualization, as has previously been suggested to be necessary to build research capacity in sub-Saharan Africa and in LMIC in general [7, 9, 13, 64, 69, 70]. The complexities of comparing cross-cultural data based on self-reports of subjective well-being, especially if it involves the translation and adaption of scales, are well described in the literature [71–73]. If authors chose to present conclusions based on assumed comparability of measurements, it has been recommended to report on measurement invariance issues in clear terms and point out any potential pitfalls in regard to the interpretation of results to the reader, e.g. to policy-makers [74]. We suggest future studies adopt this as good practice.

This review has implications for program developers. To further improve and develop adequate child and adolescent mental health care, interventions should also be delivered in educational, health and social care, and community settings. Mental health should be an integral aspect of programs related to community empowerment, poverty reduction, HIV/AIDS prevention, and reproductive and sexual health. Mental health prevention and promotion programs have been implemented effectively in LMIC school settings [75], but it also needs adequate programs to support hard-to-reach groups, such as adolescents that are ‘out of school’, and homeless youth [76].

### Strengths and limitations

Blind selection of studies and a comprehensive search strategy that enabled the capture of a large number of studies were strengths of this review. This review has several limitations that should be acknowledged when interpreting the results. We only explicitly named South Africa and Zambia in our search terms and this may have may have resulted in finding more studies from South Africa and Zambia. Adapting the search terms to include all 48 sub-Saharan countries would have definitely improved the sensitivity and scope of our search. While we conducted an extensive search for studies, our search did not cover grey literature. Due to limited resources available for translation, we also did not include studies published in peer-review non-English language journals. This quite possibly led to an underrepresentation of other regions, for example, Francophone countries in West Africa are not well represented in this review. Several additional studies were identified through handsearching the reference lists of journal articles, suggesting a limitation in the search strategy. The authors conducting the search did not have access to specific Africa databases and we are aware that regional-based databases would have given further insight into the topic and improved the sensitivity of the search. A number of studies reporting data from the more recent GSHS studies were not found in the search and we suggest the recent systematic review by Quarshie, Waterman and House [69] for a more comprehensive overview of studies reporting on self-harm with suicidal and non-suicidal intent in young people in sub-Saharan Africa. The heterogeneity across studies was high, attributed to by differences in methodology, community sample characteristics, type of screening and diagnostic tools used, translation and validation of tools, how and by whom assessments were performed and other unexplained variations. In addition, many studies did not report on the number of participants who did not complete the questionnaires or on the number of potential participants who were approached but were not recruited into the study.

## Conclusion

This systematic review draws attention to the gaps that remain in knowledge regarding the burden of mental disorders among youth in sub-Saharan Africa. Although there is wide variation in estimates of common mental disorders and symptoms in youth on the continent, it is evident that rates are high with and require concerted attention at both clinical and policy level.

## Supporting information

S1 Table Overview of the studies included. (TIFF)

S2 Table Critical appraisal of included studies according to Joanna Briggs Institute. (TIFF)

S3 Table Overview of reported associated factors. (TIFF)

S4 Table Search terms for search strategy. (TIFF)

S5 Table PRISMA checklist. (TIFF)

S6 Table Minimal data set. (TIFF)

## References

[1] Patel V, Flisher AJ, Nikapota A, Malhotra S. Promoting child and adolescent mental health in low and middle income countries. J Child Psychol Psychiatry. 2008;49: 313–334. 10.1111/j.1469-7610.2007.01824.x 18093112

[2] Burns J. Psychosocial determinants of mental disorders. In: Burns J, Louw R, editors. Textbook of Psychiatry for Southern Africa. Cape Town: Oxford University Press Southern Africa; 2016. pp. 72–90.

[3] Erskine HE, Moffitt TE, Copeland WE, Costello EJ, Ferrari AJ, Patton G, et al. A heavy burden on young minds: the global burden of mental and substance use disorders in children and youth. Psychol Med. 2015;45: 1551–1563. 10.1017/S0033291714002888 25534496 PMC5922255

[4] UNICEF. Adolescent Demographics. In: UNICEF DATA [Internet]. Oct 2019 [cited 2 Sep 2020]. https://data.unicef.org/topic/adolescents/demographics/

[5] Kieling C, Baker-Henningham H, Belfer M, Conti G, Ertem I, Omigbodun O, et al. Child and adolescent mental health worldwide: evidence for action. The Lancet. 2011;378: 1515–1525. 10.1016/S0140-6736(11)60827-1 22008427

[6] Erskine HE, Baxter AJ, Patton G, Moffitt TE, Patel V, Whiteford HA, et al. The global coverage of prevalence data for mental disorders in children and adolescents. Epidemiol Psychiatr Sci. 2017;26: 395–402. 10.1017/S2045796015001158 26786507 PMC6998634

[7] Atilola O. Cross-cultural child and adolescent psychiatry research in developing countries. Glob Ment Health. 2015;2. 10.1017/gmh.2015.8 28596853 PMC5269637

[8] Lwidiko A, Kibusi SM, Nyundo A, Mpondo BCT. Association between HIV status and depressive symptoms among children and adolescents in the Southern Highlands Zone, Tanzania: A case-control study. PloS One. 2018;13: e0193145. 10.1371/journal.pone.0193145 29470512 PMC5823441

[9] Omigbodun OO, Belfer ML. Building research capacity for child and adolescent mental health in Africa. Child Adolesc Psychiatry Ment Health. 2016;10. 10.1186/s13034-016-0119-2 27594899 PMC5009699

[10] World Health Organization. World health statistics overview 2019: monitoring health for the SDGs, sustainable development goals. Geneva: World Health Organization; 2019. https://apps.who.int/iris/handle/10665/311696

[11] Silva SA, Silva SU, Ronca DB, Gonçalves VSS, Dutra ES, Carvalho KMB. Common mental disorders prevalence in adolescents: A systematic review and meta-analyses. PLoS ONE. 2020;15. 10.1371/journal.pone.0232007 32324835 PMC7179924

[12] Polanczyk GV, Salum GA, Sugaya LS, Caye A, Rohde LA. Annual Research Review: A meta-analysis of the worldwide prevalence of mental disorders in children and adolescents. J Child Psychol Psychiatry. 2015;56: 345–365. 10.1111/jcpp.12381 25649325

[13] Cortina MA. Prevalence of Child Mental Health Problems in Sub-Saharan Africa: A Systematic Review. Arch Pediatr Adolesc Med. 2012;166: 276. 10.1001/archpediatrics.2011.592 22393184

[14] Lahti M, Groen G, Mwape L, Korhonen J, Breet E, Chapima F, et al. Design and Development Process of a Youth Depression Screening m-Health Application for Primary Health Care Workers in South Africa and Zambia: An Overview of the MEGA Project. Issues Ment Health Nurs. 2019; 1–7. 10.1080/01612840.2019.1604919 31225763

[15] Dessauvagie AS, Jörns-Presentati A, Napp A-K, Stein DJ, Jonker D, Breet E, et al. The prevalence of mental health problems in sub-Saharan adolescents living with HIV: a systematic review. Glob Ment Health. 2020;7: e29. 10.1017/gmh.2020.18 33489245 PMC7786273

[16] Munn Z, Moola S, Lisy K, Riitano D, Tufanaru C. Methodological guidance for systematic reviews of observational epidemiological studies reporting prevalence and cumulative incidence data: Int J Evid Based Healthc. 2015;13: 147–153. 10.1097/XEB.0000000000000054 26317388

[17] The World Bank. World Bank Country and Lending Groups—World Bank Data Help Desk. 2020 [cited 1 Sep 2020]. https://datahelpdesk.worldbank.org/knowledgebase/articles/906519-world-bank-country-and-lending-groups

[18] Moher D, Liberati A, Tetzlaff J, Altman DG, The PRISMA Group. Preferred Reporting Items for Systematic Reviews and Meta-Analyses: The PRISMA Statement. PLoS Med. 2009;6: e1000097. 10.1371/journal.pmed.1000097 19621072 PMC2707599

[19] Munn Z, Moola S, Riitano D, Lisy K. The development of a critical appraisal tool for use in systematic reviews addressing questions of prevalence. Int J Health Policy Manag. 2014;3: 123–128. 10.15171/ijhpm.2014.71 25197676 PMC4154549

[20] Aromataris E, Fernandez R, Godfrey CM, Holly C, Khalil H, Tungpunkom P. Summarizing systematic reviews: methodological development, conduct and reporting of an umbrella review approach. Int J Evid Based Healthc. 2015;13: 132–140. 10.1097/XEB.0000000000000055 26360830

[21] Adeniyi AF, Okafor NC, Adeniyi CY. Depression and physical activity in a sample of nigerian adolescents: levels, relationships and predictors. Child Adolesc Psychiatry Ment Health. 2011;5. 10.1186/1753-2000-5-16 21569581 PMC3117803

[22] Bach JM, Louw D. Depression and exposure to violence among Venda and Northern Sotho adolescents in South Africa. Afr J Psychiatry. 2010;13: 25–35. 10.4314/ajpsy.v13i1.53426 20428596

[23] Berhane Y, Canavan CR, Darling AM, Sudfeld CR, Vuai S, Adanu R, et al. The age of opportunity: prevalence of key risk factors among adolescents 10–19 years of age in nine communities in sub-Saharan Africa. Trop Med Int Health. 2020;25: 15–32. 10.1111/tmi.13339 31698531

[24] Brown DW, Riley L, Butchart A, Kann L. Bullying among youth from eight African countries and associations with adverse health behaviors. Pediatr Health. 2008;2: 289–299. 10.2217/17455111.2.3.289

[25] Buckley J, Otwombe K, Joyce C, Leshabane G, Hornschuh S, Hlongwane K, et al. Mental Health of Adolescents in the Era of Antiretroviral Therapy: Is There a Difference Between HIV-Infected and Uninfected Youth in South Africa? J Adolesc Health. 2020;67: 76–83. 10.1016/j.jadohealth.2020.01.010 32269000

[26] Cheng Y, Li X, Lou C, Sonenstein FL, Kalamar A, Jejeebhoy S, et al. The Association Between Social Support and Mental Health Among Vulnerable Adolescents in Five Cities: Findings From the Study of the Well-Being of Adolescents in Vulnerable Environments. J Adolesc Health. 2014;55: S31–S38. 10.1016/j.jadohealth.2014.08.020 25454000

[27] Closson K, Dietrich JJ, Nkala B, Musuku A, Cui Z, Chia J, et al. Prevalence, type, and correlates of trauma exposure among adolescent men and women in Soweto, South Africa: implications for HIV prevention. BMC Public Health. 2016;16: 1191. 10.1186/s12889-016-3832-0 27884181 PMC5123224

[28] Cluver L, Orkin M. Cumulative risk and AIDS-orphanhood: Interactions of stigma, bullying and poverty on child mental health in South Africa. Soc Sci Med. 2009;69: 1186–1193. 10.1016/j.socscimed.2009.07.033 19713022

[29] Cluver L, Orkin M, Boyes ME, Sherr L, Makasi D, Nikelo J. Pathways from parental AIDS to child psychological, educational and sexual risk: developing an empirically-based interactive theoretical model. Soc Sci Med 1982. 2013;87: 185–193. 10.1016/j.socscimed.2013.03.028 23631794

[30] Cluver L, Orkin M, Boyes ME, Sherr L. Child and Adolescent Suicide Attempts, Suicidal Behavior, and Adverse Childhood Experiences in South Africa: A Prospective Study. J Adolesc Health. 2015;57: 52–59. 10.1016/j.jadohealth.2015.03.001 25936843

[31] Cortina MA, Fazel M, Hlungwani TM, Kahn K, Tollman S, Cortina-Borja M, et al. Childhood psychological problems in school settings in rural Southern Africa. PloS One. 2013;8: e65041. 10.1371/journal.pone.0065041 23776443 PMC3680478

[32] Doku PN, Minnis H. Multi-informant perspective on psychological distress among Ghanaian orphans and vulnerable children within the context of HIV/AIDS. Psychol Med. 2016;46: 2329–2336. 10.1017/S0033291716000829 27270076

[33] Fatiregun AA, Kumapayi TE. Prevalence and correlates of depressive symptoms among in-school adolescents in a rural district in southwest Nigeria. J Adolesc. 2014;37: 197–203. 10.1016/j.adolescence.2013.12.003 24439625

[34] Gage AJ. Association of Child Marriage With Suicidal Thoughts and Attempts Among Adolescent Girls in Ethiopia. J Adolesc Health. 2013;52: 654–656. 10.1016/j.jadohealth.2012.12.007 23433537

[35] Goin DE, Pearson RM, Craske MG, Stein A, Pettifor A, Lippman SA, et al. Depression and Incident HIV in Adolescent Girls and Young Women in HIV Prevention Trials Network 068: Targets for Prevention and Mediating Factors. Am J Epidemiol. 2020;189: 422–432. 10.1093/aje/kwz238 31667490 PMC7306677

[36] Ismayilova L, Gaveras E, Blum A, To-Camier A, Nanema R. Maltreatment and Mental Health Outcomes among Ultra-Poor Children in Burkina Faso: A Latent Class Analysis. PloS One. 2016;11: e0164790. 10.1371/journal.pone.0164790 27764155 PMC5072722

[37] Kinyanda E, Kizza R, Levin J, Ndyanabangi S, Abbo C. Adolescent Suicidality as Seen in Rural Northeastern Uganda: Prevalence and Risk Factors. Crisis. 2011;32: 43–51. 10.1027/0227-5910/a000059 21371970

[38] Kuringe E, Materu J, Nyato D, Majani E, Ngeni F, Shao A, et al. Prevalence and correlates of depression and anxiety symptoms among out-of-school adolescent girls and young women in Tanzania: A cross-sectional study. Francis JM, editor. PLOS ONE. 2019;14: e0221053. 10.1371/journal.pone.0221053 31419238 PMC6697336

[39] Kyohangirwe L, Okello E, Namuli JD, Ndeezi G, Kinyanda E. Prevalence and factors associated with major depressive disorder among adolescents attending a primary care facility in Kampala, Uganda. Trop Doct. 2020;50: 30–36. 10.1177/0049475519879586 31594531

[40] Lachman JM, Cluver LD, Boyes ME, Kuo C, Casale M. Positive parenting for positive parents: HIV/AIDS, poverty, caregiver depression, child behavior, and parenting in South Africa. AIDS Care. 2014;26: 304–313. 10.1080/09540121.2013.825368 23930647 PMC3922642

[41] Mashego T-AB, Madu SN. Suicide-Related Behaviours among Secondary School Adolescents in the Welkom and Bethlehem Areas of the Free State Province (South Africa). South Afr J Psychol. 2009;39: 489–497. 10.1177/008124630903900410

[42] Nkuba M, Hermenau K, Goessmann K, Hecker T. Mental health problems and their association to violence and maltreatment in a nationally representative sample of Tanzanian secondary school students. Soc Psychiatry Psychiatr Epidemiol. 2018;53: 699–707. 10.1007/s00127-018-1511-4 29651620

[43] Nyundo A, Manu A, Regan M, Ismail A, Chukwu A, Dessie Y, et al. Factors associated with depressive symptoms and suicidal ideation and behaviours amongst sub-Saharan African adolescents aged 10–19 years: cross-sectional study. Trop Med Int Health. 2020;25: 54–69. 10.1111/tmi.13336 31698526

[44] Omigbodun O, Dogra N, Esan O, Adedokun B. Prevalence and Correlates of Suicidal Behaviour Among Adolescents in Southwest Nigeria. Int J Soc Psychiatry. 2008;54: 34–46. 10.1177/0020764007078360 18309757

[45] Osborn TL, Venturo-Conerly KE, Wasil AR, Schleider JL, Weisz JR. Depression and Anxiety Symptoms, Social Support, and Demographic Factors Among Kenyan High School Students. J Child Fam Stud. 2020;29: 1432–1443. 10.1007/s10826-019-01646-8

[46] Page RM, Saumweber J, Hall PC, Crookston BT, West JH. Multi-country, cross-national comparison of youth suicide ideation: Findings from Global School-based Health Surveys. Sch Psychol Int. 2013;34: 540–555.

[47] Peltzer K. Prevalence and correlates of substance use among school children in six African countries. Breslau Abiodun B, Bruvold, Challier, DuRant, Faeh, Geckova, Godeau, Grant, Hanna, King, Kokkevi, Lai, Nutbeam, Peltzer, Resnick, Sale, Schmid, Yang, editor. Int J Psychol. 2009;44: 378–386. 10.1080/00207590802511742 22029616

[48] Shangani S, Operario D, Genberg B, Kirwa K, Midoun M, Atwoli L, et al. Unconditional government cash transfers in support of orphaned and vulnerable adolescents in western Kenya: Is there an association with psychological wellbeing? PloS One. 2017;12: e0178076. 10.1371/journal.pone.0178076 28562627 PMC5451046

[49] Stansfeld SA, Rothon C, Das-Munshi J, Mathews C, Adams A, Clark C, et al. Exposure to violence and mental health of adolescents: South African Health and Well-being Study. BJPsych Open. 2017;3: 257–264. 10.1192/bjpo.bp.117.004861 29093828 PMC5643877

[50] Strydom MAA, Pretorius PJ, Joubert G. Depression and anxiety among Grade 11 and 12 learners attending schools in central Bloemfontein. South Afr J Psychiatry. 2012;18. 10.7196/sajp.356

[51] Shilubane HN, Ruiter RA, van den Borne B, Sewpaul R, James S, Reddy PS. Suicide and related health risk behaviours among school learners in South Africa: results from the 2002 and 2008 national youth risk behaviour surveys. BMC Public Health. 2013;13: 926. 10.1186/1471-2458-13-926 24093214 PMC3851142

[52] Suliman S, Mkabile SG, Fincham DS, Ahmed R, Stein DJ, Seedat S. Cumulative effect of multiple trauma on symptoms of posttraumatic stress disorder, anxiety, and depression in adolescents. Compr Psychiatry. 2009;50: 121–127. 10.1016/j.comppsych.2008.06.006 19216888

[53] Thurman TR, Nice J, Taylor TM, Luckett B. Mitigating depression among orphaned and vulnerable adolescents: a randomized controlled trial of interpersonal psychotherapy for groups in South Africa. Child Adolesc Ment Health. 2017;22: 224–231. 10.1111/camh.12241 32680417

[54] van Renen LJ, Wild LG. Family functioning and suicidal ideation/behaviour in adolescents: a pilot study. J Child Adolesc Ment Health. 2008;20: 111–121. 10.2989/JCAMH.2008.20.2.7.690 25865589

[55] Ward CL, Artz L, Leoschut L, Kassanjee R, Burton P. Sexual violence against children in South Africa: a nationally representative cross-sectional study of prevalence and correlates. Lancet Glob Health. 2018;6: e460–e468. 10.1016/S2214-109X(18)30060-3 29530424

[56] Vawda N. The prevalence of suicidal behaviour and associated risk factors in grade 8 learners in Durban. South Afr Fam Pract. 2014;56: 37–42. 10.1080/20786204.2014.10844581

[57] Zietz S, Iritani BJ, Otieno FA, Ongili BO, Odongo FS, Rennie S, et al. Suicide behaviour among adolescents in a high HIV prevalence region of western Kenya: A mixed-methods study. Glob Public Health. 2020; 1–15. 10.1080/17441692.2020.1782964 32567992 PMC7752827

[58] Hoy D, Brooks P, Woolf A, Blyth F, March L, Bain C, et al. Assessing risk of bias in prevalence studies: modification of an existing tool and evidence of interrater agreement. J Clin Epidemiol. 2012;65: 934–939. 10.1016/j.jclinepi.2011.11.014 22742910

[59] Patel V, Flisher AJ, Hetrick S, McGorry P. Mental health of young people: a global public-health challenge. Lancet Lond Engl. 2007;369: 1302–1313. 10.1016/S0140-6736(07)60368-7 17434406

[60] McKinnon B, Gariépy G, Sentenac M, Elgar FJ. Adolescent suicidal behaviours in 32 low- and middle-income countries [Comportements suicidaires des adolescents dans 32 pays à revenu faible et intermédiaire] [Conductas suicidas de los adolescentes en 32 países con ingresos bajos y medios]. Bull World Health Organ. 2016;94: 340–350F. 10.2471/BLT.15.163295 27147764 PMC4850530

[61] Yatham S, Sivathasan S, Yoon R, da Silva TL, Ravindran AV. Depression, anxiety, and post-traumatic stress disorder among youth in low and middle income countries: A review of prevalence and treatment interventions. Asian J Psychiatry. 2017 [cited 18 Apr 2018]. 10.1016/j.ajp.2017.10.029 29117922

[62] Zarafshan H, Mohammadi M-R, Salmanian M. Prevalence of Anxiety Disorders among Children and Adolescents in Iran: A Systematic Review. Iran J Psychiatry. 2015;10: 1–7. 26005473 PMC4434422

[63] Atilola O. Where Lies the Risk? An Ecological Approach to Understanding Child Mental Health Risk and Vulnerabilities in Sub-Saharan Africa. In: Psychiatry Journal [Internet]. 2014 [cited 12 Jun 2018]. 10.1155/2014/698348 24834431 PMC4009193

[64] Ng QX, Soh AYS, Loke W, Venkatanarayanan N, Lim DY, Yeo W-S. Systematic review with meta-analysis: The association between post-traumatic stress disorder and irritable bowel syndrome. J Gastroenterol Hepatol. 2019;34: 68–73. 10.1111/jgh.14446 30144372

[65] Kessler RC, Amminger GP, Aguilar-Gaxiola S, Alonso J, Lee S, Ustun TB. Age of onset of mental disorders: a review of recent literature: Curr Opin Psychiatry. 2007;20: 359–364. 10.1097/YCO.0b013e32816ebc8c 17551351 PMC1925038

[66] Matos AP. The relationship between internalizing and externalizing problemsin adolescence: does gender make a difference? Can Int J Soc Sci Educ. 2017; 45–63. Available: https://estudogeral.sib.uc.pt/handle/10316/47157

[67] Fisher JR, Cabral de Mello M. Using the World Health Organization’s 4S-Framework to Strengthen National Strategies, Policies and Services to Address Mental Health Problems in Adolescents in Resource-Constrained Settings. Int J Ment Health Syst. 2011;5: 23. 10.1186/1752-4458-5-23 21923901 PMC3182992

[68] Stein DJ, He Y, Phillips A, Sahakian BJ, Williams J, Patel V. Global mental health and neuroscience: potential synergies. Lancet Psychiatry. 2015;2: 178–185. 10.1016/S2215-0366(15)00014-0 26359754

[69] Quarshie EN-B, Waterman MG, House AO. Self-harm with suicidal and non-suicidal intent in young people in sub-Saharan Africa: a systematic review. BMC Psychiatry. 2020;20: 234. 10.1186/s12888-020-02587-z 32408896 PMC7222461

[70] Stevanovic D, Jafari P, Knez R, Franic T, Atilola O, Davidovic N, et al. Can we really use available scales for child and adolescent psychopathology across cultures? A systematic review of cross-cultural measurement invariance data. Transcult Psychiatry. 2017;54: 125–152. 10.1177/1363461516689215 28157447

[71] Borsboom D. When does measurement invariance matter? Med Care. 2006;44: S176–S181. 10.1097/01.mlr.0000245143.08679.cc 17060825

[72] Chen FF. What happens if we compare chopsticks with forks? The impact of making inappropriate comparisons in cross-cultural research. J Pers Soc Psychol. 2008;95: 1005–1018. 10.1037/a0013193 18954190

[73] Sass DA. Testing Measurement Invariance and Comparing Latent Factor Means Within a Confirmatory Factor Analysis Framework. J Psychoeduc Assess. 2011;29: 347–363. 10.1177/0734282911406661

[74] Avvisati F, Le Donné N, Paccagnella M. A meeting report: cross-cultural comparability of questionnaire measures in large-scale international surveys. Meas Instrum Soc Sci. 2019;1: 8. 10.1186/s42409-019-0010-z

[75] Barry MM, Clarke AM, Jenkins R, Patel V. A systematic review of the effectiveness of mental health promotion interventions for young people in low and middle income countries. BMC Public Health. 2013;13: 835. 10.1186/1471-2458-13-835 24025155 PMC3848687

[76] Asante KO, Meyer-Weitz A. Prevalence and predictors of suicidal ideations and attempts among homeless children and adolescents in Ghana. J Child Adolesc Ment Health. 2017;29: 27–37. 10.2989/17280583.2017.1287708 28403747

