# Supplementary figures and images for: The prevalence of mental health problems in sub-Saharan adolescents: A systematic review
Source: PLoS One. 2021 May 14;16(5):e0251689. doi: 10.1371/journal.pone.0251689 (PMC8121357; doi:10.1371/journal.pone.0251689)

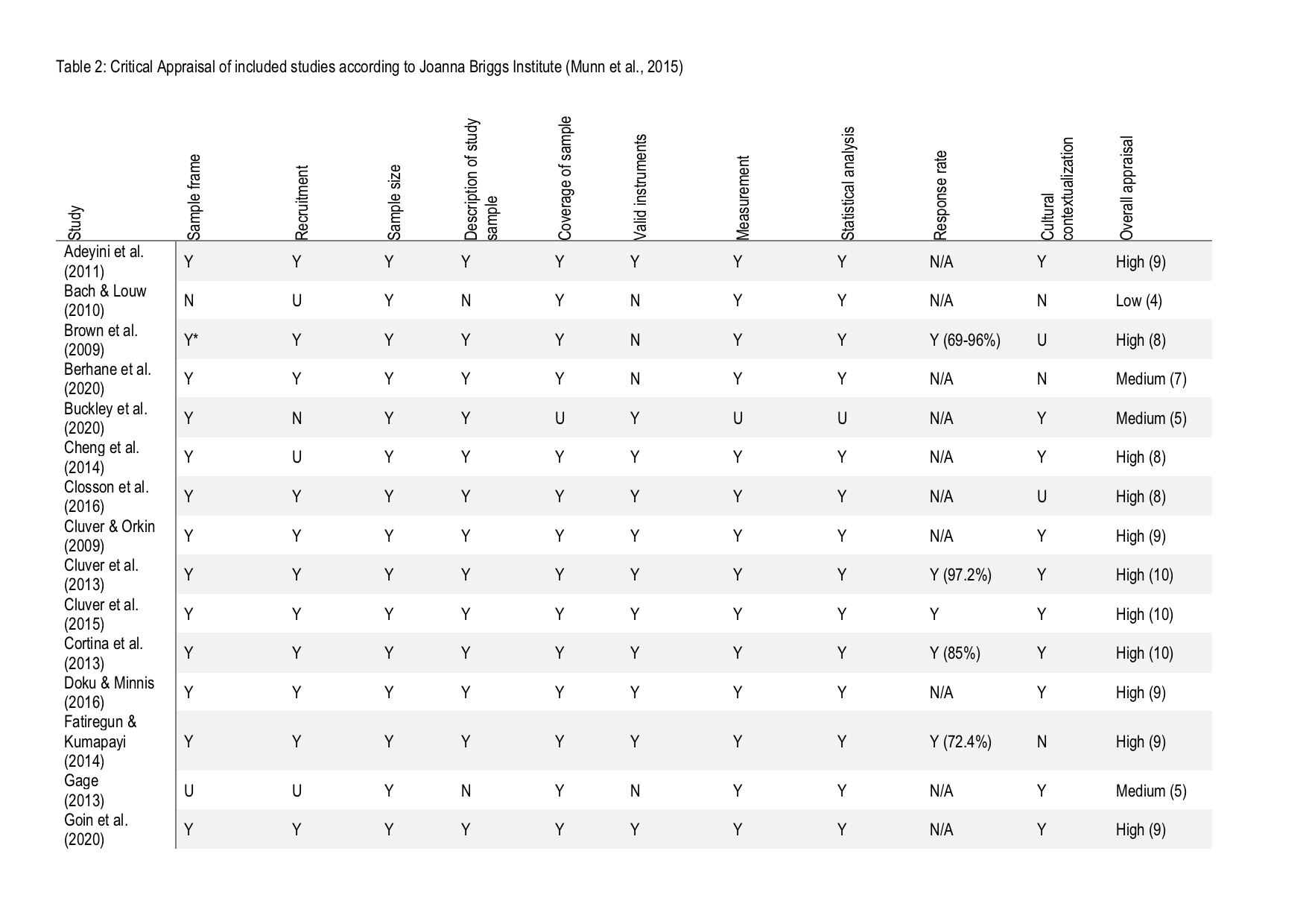

Supplement: S2 Table — (TIFF) [file pone.0251689.s002.tiff]

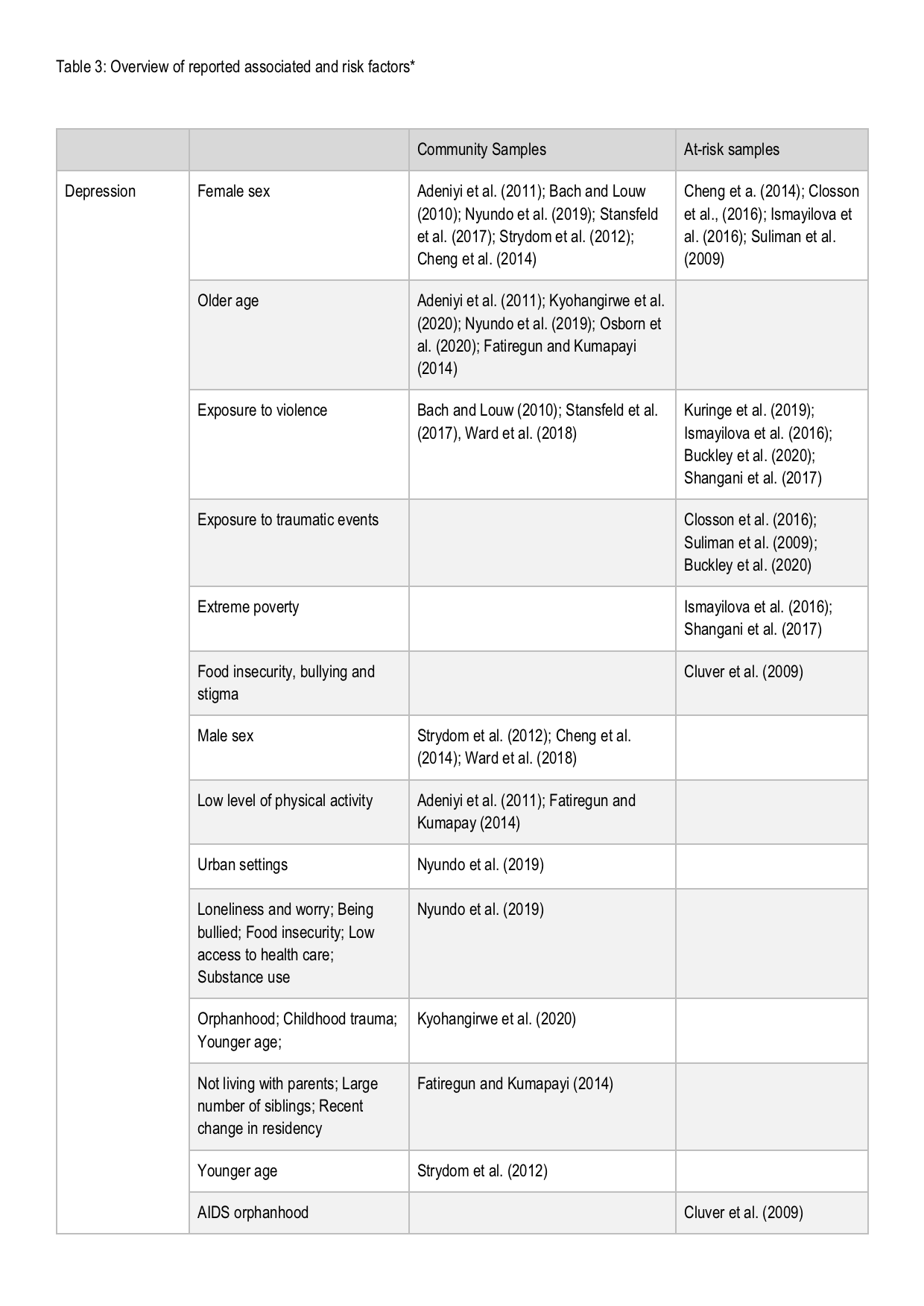

Supplement: S3 Table — (TIFF) [file pone.0251689.s003.tiff]

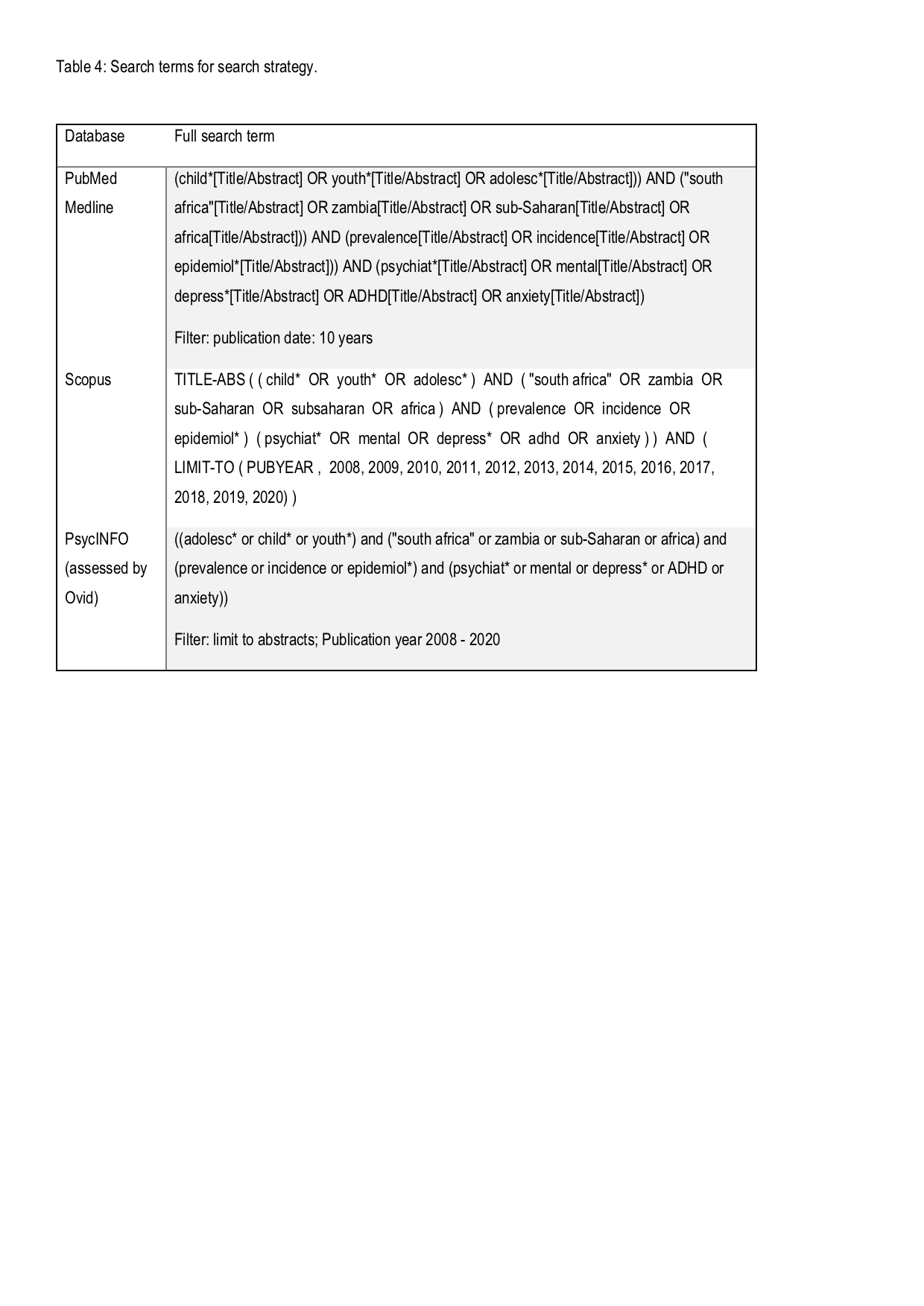

Supplement: S4 Table — (TIFF) [file pone.0251689.s004.tiff]

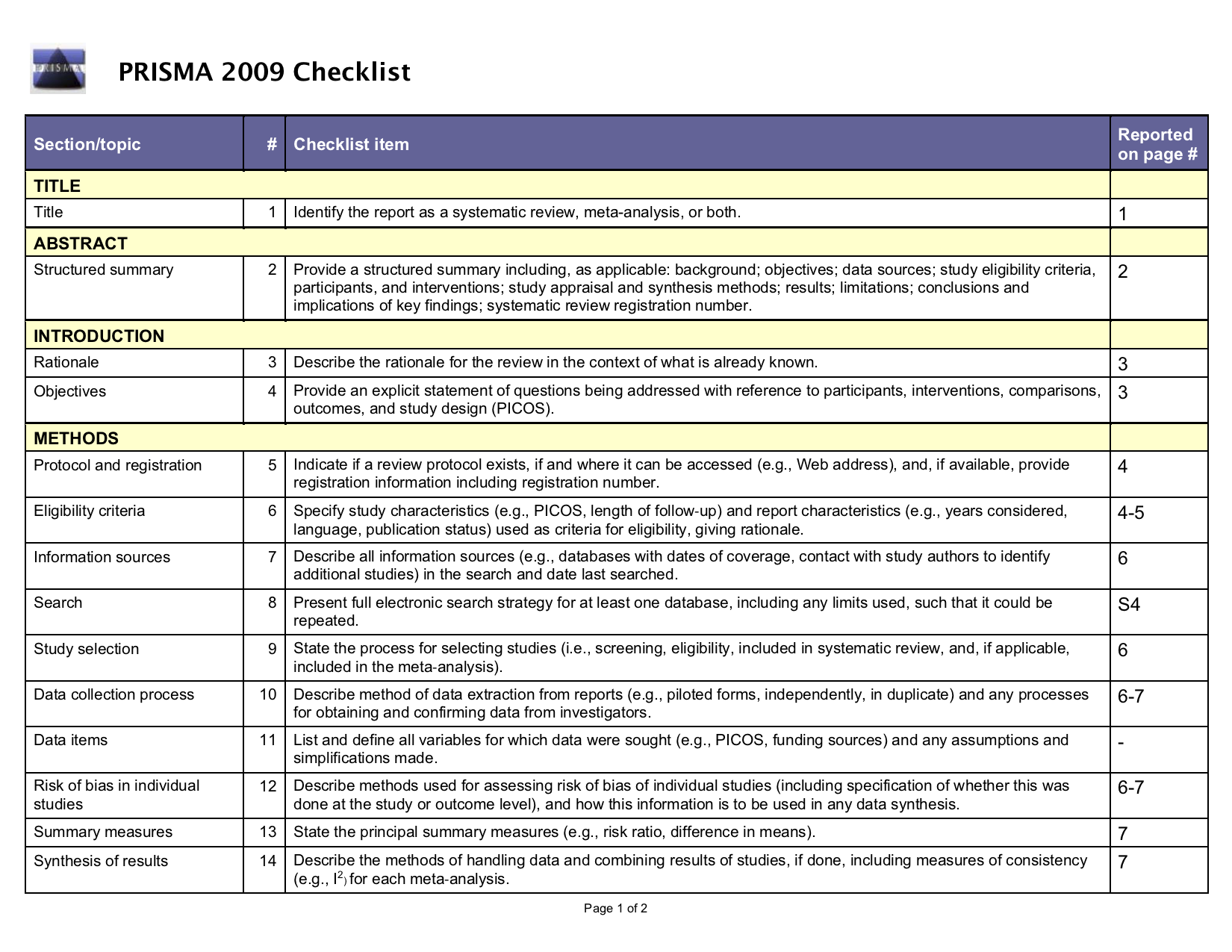

Supplement: S5 Table — (TIFF) [file pone.0251689.s005.tiff]

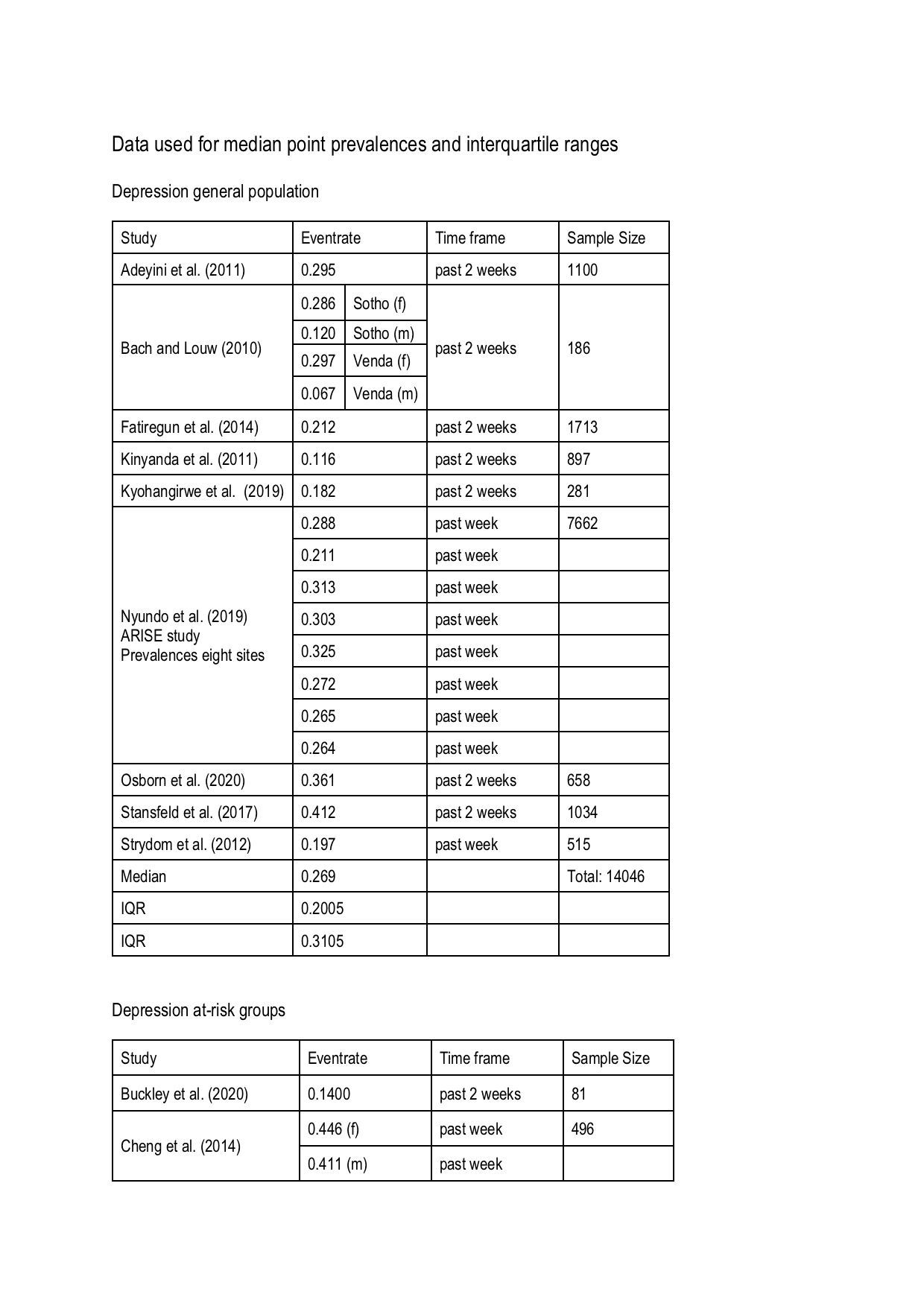

Supplement: S6 Table — (TIFF) [file pone.0251689.s006.tiff]
